# Pathophysiological aspects of neuro-COVID

**DOI:** 10.1590/0037-8682-0381-2021

**Published:** 2022-02-25

**Authors:** Josef Finsterer, Fulvio A Scorza, Ana C Fiorini

**Affiliations:** 1 Messerli Institute, Neurology & Neurophysiology Center, Vienna, Austria.; 2 Universidade Federal de São Paulo, Escola Paulista de Medicina, Disciplina de Neurociência, São Paulo, SP, Brasil.; 3 Pontifícia Universidade Católica de São Paulo, Departamento de Fonoaudiologia, São Paulo, SP, Brasil.; 4 Universidade Federal de São Paulo, Escola Paulista de Medicina, São Paulo, SP, Brasil.

We read the review article by Puccioni-Sohler et al. about the diagnostic management and pathophysiological mechanisms responsible for neuro-COVID with great interest^1^. The review concluded that understanding the neurological manifestations and pathophysiology of SARS-CoV-2 infections is a prerequisite for correct diagnosis and adequate treatment^1^. The review is appealing but has several limitations that raise the following comments and concerns.

Only some of the pathophysiological mechanisms explaining neuro-COVID were discussed. A pathophysiological mechanism explaining SARS-CoV-2-associated central nervous system (CNS) disease not acknowledged in the review is cardiac involvement. Cardiac manifestations of a SARS-CoV-2 infection include myocarditis, pericarditis, or endocarditis. These conditions may be complicated by arterial hypertension, supraventricular or ventricular arrhythmias, Takotsubo syndrome (TTS), heart failure, or systolic dysfunction. Arrhythmias or heart failure may be accompanied by cerebral hypoperfusion and hypoxia, thromboembolism, or impaired autoregulation of the cerebral perfusion. Accordingly, ischemic stroke, intracerebral bleeding, posterior reversible encephalopathy syndrome, or cerebral vasoconstriction syndrome may ensue. Myocarditis or heart failure may be caused by the direct invasion of cardiomyocytes or endothelial cells of coronary arteries by the virus. Arterial hypertension may be caused by autonomic dysfunction. TTS may be triggered by psychological or physical stress from the viral infection and its treatment.

A second pathophysiological mechanism of neuro-COVID, particularly in patients with severe COVID-19 requiring intensive care unit (ICU) treatment, that was not addressed, are the complications of ICU treatment. Patients with severe COVID-19 requiring short- or long-term ICU treatment may develop critical illness neuropathy or critical illness myopathy. ICU treatment may be additionally complicated by pressure palsies or compartment syndrome. In case of superinfection due to SARS-CoV-2-induced immunosuppression and the development of sepsis, patients may experience septic encephalopathy. Long-term ventilator support may be complicated by cerebral hypoxia.

A third pathophysiological mechanism explaining manifestations of neuro-COVID not addressed in the review is neuro- or myotoxicity of anti-COVID-19 drugs. From several compounds frequently applied in the therapeutic management of moderate or severe COVID-19, it is well known that they occasionally exhibit adverse reactions, which may be responsible for neurological compromise. Potentially neurotoxic drugs applied in COVID-19 patients include daptomycin, linezolid, lopinavir, ritonavir, hydroxychloroquine, cisatracurium, clindamycin, tocilizumab, and glucocorticoids^2^. Potentially myotoxic drugs administered to COVID-19 patients include chloroquine (occasionally causes myopathy or myasthenia)^3^, remdesivir/lopinavir (occasionally causes rhabdomyolysis)^4^, azithromycin (occasionally causes myasthenia or myasthenic crisis)^5^, tocilizumab (occasionally causes pyomyositis)^6^, and steroids (occasionally cause mitochondrial myopathy)^7^.

Overall, the pathophysiological spectrum of neuro-COVID is broader than anticipated. SARS-CoV-2-associated cardiac involvement, complications of ICU treatment, and toxicity of anti-COVID-19 drugs may contribute to the understanding of the pathophysiological background of neuro-COVID.
